# Finger-Counting-Based Gesture Recognition within Cars Using Impulse Radar with Convolutional Neural Network

**DOI:** 10.3390/s19061429

**Published:** 2019-03-23

**Authors:** Shahzad Ahmed, Faheem Khan, Asim Ghaffar, Farhan Hussain, Sung Ho Cho

**Affiliations:** 1 Department of Electronics and Computer Engineering, Hanyang University, 222 Wangsimini-ro, Seongdong-gu, Seoul 04763, Korea; shahzad1@hanyang.ac.kr (S.A.); faheemkhan@hanyang.ac.kr (F.K.); asimghaffar@hanyang.ac.kr (A.G.); 2 College of Electrical and Mechanical Engineering, National University of Science and Technology, Islamabad 44000, Pakistan; farhan.hussain@ceme.nust.edu.pk

**Keywords:** impulse radar sensor, gesture recognition, finger counting, deep learning classifier, convolutional neural network

## Abstract

The diversion of a driver’s attention from driving can be catastrophic. Given that conventional button- and touch-based interfaces may distract the driver, developing novel distraction-free interfaces for the various devices present in cars has becomes necessary. Hand gesture recognition may provide an alternative interface inside cars. Given that cars are the targeted application area, we determined the optimal location for the radar sensor, so that the signal reflected from the driver’s hand during gesturing is unaffected by interference from the motion of the driver’s body or other motions within the car. We implemented a Convolutional Neural Network-based technique to recognize the finger-counting-based hand gestures using an Impulse Radio (IR) radar sensor. The accuracy of the proposed method was sufficiently high for real-world applications.

## 1. Introduction

During the last century, cars and other vehicles were merely considered a means of transportation. However, of late, cars are becoming highly advanced machines that provide a lot of additional benefits along with transportation. In fact, cars can now be referred to as “offices on the move” [1] or “personal communication centers” [2] with additional controls and functionalities. Producing cars now involves more than simply designing a space for transportation and delivery [3]. The development of convenient user interfaces for drivers is very important because vehicular safety depends on ensuring that the driver’s focus remains on the road. Currently, different conventional interfaces are used within cars to control the various electrical and electronic devices present, such as button-based and touch-based interfaces. The disadvantages of these conventional interfaces are that they distract the driver from the primary job of monitoring the road and can thus cause car crashes. Speech recognition is another solution. However, changes in the voice tone or speech clarity can cause glitches, as the speech in this case would be translated as unrecognized words or acronyms [4]. Moreover, speech recognition is also dependent on several operational and environmental factors, which may reduce the speed and accuracy of recognition. Gesture-based interfaces can be useful as they do not distract the driver from monitoring the road because they do not require visual attention. Further, radar-based gesture recognition is not affected by environmental conditions such as the lighting, humidity, or temperature [5].

Gesture classification is a hot research topic these days. Finger-counting-based gestures may provide an easy human-computer interface (HCI) that may be suitable for use in a range of applications such as electronic device control within vehicles, television remote control, and indoor electrical device control. Currently, some of the widely used sensors for gesture recognition include cameras [6,7], radio-frequency identification systems [8], and data gloves [9]. Depth cameras have a high resolution, which allows them to track and recognize finger movements. In fact, researchers have been able to perform camera-based gesture recognition with an average recognition rate of 98.5% for finger counting [10]. The drawback of using cameras for gesture recognition is their poor performance in dark and highly lit environments. Another disadvantage of camera-based gesture recognition is related to the privacy of the users. Another method for gesture recognition is to use glove-based sensors. Data-glove-based methods use sensors for digitizing hand and finger motions into multi-parametric data [9]. Although the use of additional sensors for glove-based gesture recognition makes it easy to collect hand movement and configuration data, these sensors are not highly wearable and can cause discomfort to the user [11]. In contrast to the gesture recognition technologies discussed above, gesture recognition with radar has no privacy issues and is also convenient for the user because it is contactless. Further, no additional wearable device is required. Impulse radio ultra-wideband (IR-UWB) radar has the additional advantages of high-range resolution and robustness with respect to multiple paths because of its high bandwidth and low power requirement [12]. IR-UWB radar has found use in several applications such as people counting [13], the monitoring of vital signs [14], three-dimensional positioning [15], and for sensing body movements such as head rotation [16] and chest movement [17]. 

One of the promising utilization of this sensor is the hand gesture recognition-based HCI development. Ren et al. [18] have developed algorithms for gesture recognition using IR-UWB radar. Although the gesture recognition accuracy of these algorithms is high, the gestures used were simply based on the differences in the position of the hand and may not be useful in applications requiring “small” hand gestures. 

Recently, Ryu et al. [19] presented a features-based hand gesture recognition technique with significantly high recognition accuracy. Wang et al. [20] developed a radar’s range and speed based features for hand gesture recognition and developed a feature map that was fed as input to classifier. However, the gestures in these studies were also “big”. In addition, all the above-mentioned studies analyzed the input signal with one-dimensional signal-processing techniques for gesture classification; these techniques may fail when attempting to classify micro-gestures because the gesture patterns reside in the images of the reflected data, and it may be easier to classify these gestures using image-processing algorithms. In this study, we used IR-UWB radar and convolutional neural network (CNN)-based classifier to detect finger-counting gestures. 

The proposed method consists of preprocessing the radar signal and then converting the two-dimensional (2D) signal into an image. A greyscale image was saved for each sample. After completing the image transformation process, recognition is tackled as an image-classification problem. These images created against radar’s data matrix serves as an input to CNN algorithm. CNN based classifiers have proven to be the state-of-the art classifier for image processing problems such as ImageNet LSVRC-2010 contest [21]. Moreover, the CNN algorithm is capable of self-learning and can deal with the input data without any prior information and its learning capacity can be controlled by varying the depth network. Features extraction and classification is performed with CNN algorithm. CNN algorithm takes the input data in form of images, learns the features by passing the input through a serial structure of layers, and classify it based on the certain categorical information commonly referred as ground-truth-information [22]. The features extraction task in this study was performed with six hidden layered CNN architecture. 

The main contribution of this work is that it is the first one to use IR-UWB radar and radar-image-based classifier design for small gesture i.e., finger counting. To the best of our knowledge, the existing work on IR-UWB radar-based hand gesture classification so far has considered big gestures only. Further, we analyzed the radar signals as an images, contrary to the majority of the previous work [13,17,18,19], where the authors have analyzed one dimensional radar signals for feature extraction and classification. We have implemented a CNN with optimized layers by experimentation so that the optimized CCN result in maximum accuracy and minimum processing time for the defined training dataset. Moreover, to make the algorithm more robust, we normalized the magnitude of the radar data matrix using the mean and variance of the data. In this work, three volunteers were used for obtaining the training and test gesture-related data, and the overall accuracy was approximately 97%. 

The rest of the manuscript is organized as follows: Section 2 provides an overview of the theoretical background of the proposed method. Next, in Section 3, the experimental setup used to implement the proposed method is described. The obtained results are described and discussed in Section 4. Finally, Section 5 presents the conclusions of the study.

## 2. Materials and Methods 

### 2.1. System Overview

**Figure 1:**
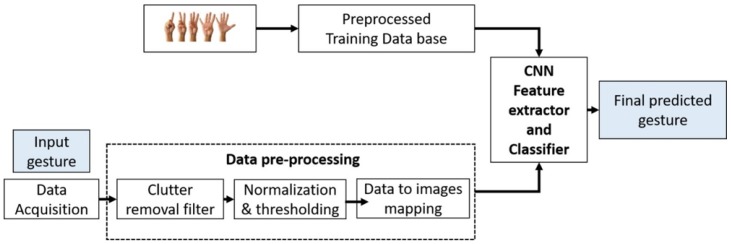
Proposed method for gesture recognition (i.e., finger counting) using convolutional neural network.

The block-diagram of the proposed method is shown in Figure 1. It primarily comprises data acquisition, data preprocessing, and CNN based training and evaluation. We installed only one IR-UWB radar within the car interior for data acquisition having a direction of propagation perpendicular to the motion of the fingers. 

### 2.2. Optimul Position of Sensor within Car

Given that the proposed gesture-based UI is intended for use by a driver inside a car, determining the optimal location for the radar sensor is very important. Driver distraction may cause accidents as well as a reduction in the vehicle speed [23,24]. Therefore, the optimal sensor location would be one that allows for a highly accessible UI that can be used without causing the driver to be distracted from the main task of monitoring the road. Moreover, the sensor should be placed at a location such that data acquisition is not affected by the other in-car motions of the driver or the other passengers. To this end, we performed simulations to evaluate three different locations within a car. 

**Figure 2:**
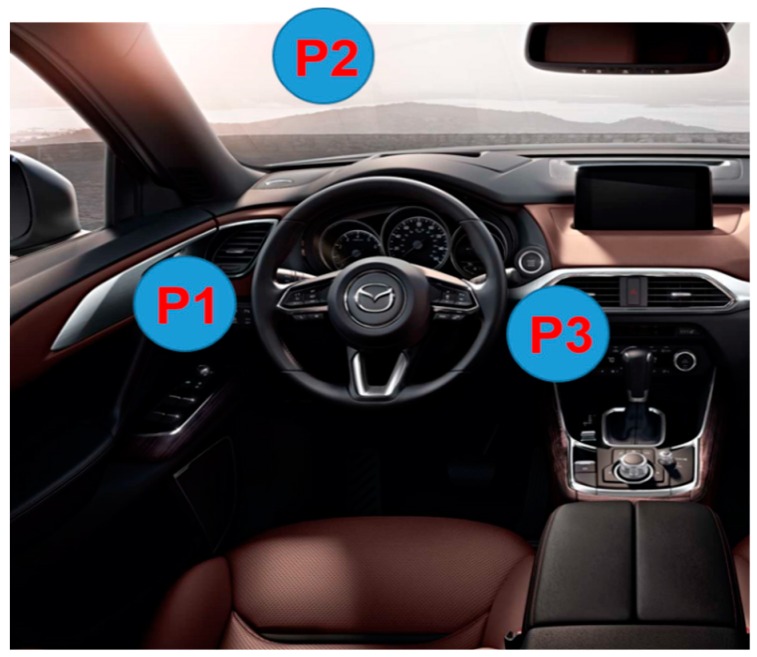
Evaluated radar locations.

Figure 2 shows the three potential locations for the radar sensor labelled as P1, P2, and P3. Position P2 provides a clear view, and the radar beam is projected directly towards the driver. However, it was observed that, in this case, the movement of the driver’s head or upper torso created unwanted artefacts. This problem can be overcome by placing the radar sensor at position P3. However, this position is exposed to the random movements that occur whenever the gear is changed. These random movements may distort the reflected signal and hence increase the probability of false detections. On the other hand, location P1 provides ease of accessibility and is not susceptible to other types of motions, making it suitable for data acquisition. 

### 2.3. Signal Preprocessing

**Figure 3:**
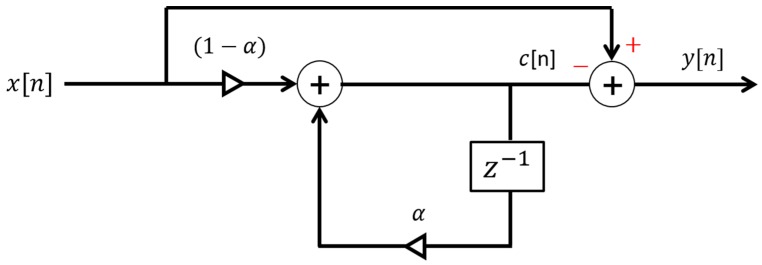
Loopback filter for clutter removal.

Usually, in the case of wireless communication systems, the received signal contains echoes of the transmitted signal, which is reflected and scattered by the different objects present in the physical medium [25]. For impulse radio, the transmitted signal is an impulse of very short duration [26]. The impulse radio transmits a series of impulses, which are widely spread in the frequency domain. These transmitted pulses, *s*[*n*], and the corresponding received signal, *x*[*n*], can be represented using an impulse train [13], as shown below:

(1) $$x\left[n\right]=\overset{N}{\underset{m=1}{\sum }}s\left[n-mN\right]$$

where ‘*m*’ is the delay between the transmitted pulses. The received wideband signal, *x*[*n*], contains information about the objects within the radar beam width. In the raw form, *x*[*n*] contains reflections from all the objects within the operational range. Some of these reflections are from the gestures of interest, while a few are reflections from static objects. Undesired radar returns are termed “clutter” [27]. Here, the radar returns from static objects within the operational range of radar are considered as clutter and need to be removed. Various filters and techniques exist for removing this information, including the Kalman filter [28], the singular value decomposition method [29], and the loopback filter [28], among others. The loopback filter is one of the most widely used filters for this purpose because of its simple structure and low computational complexity [17,28,30]. Previously, the similar filter has also been used for background subtraction in monitoring the respiratory activities using UWB radars [17]. The structure of the loopback filter is shown in Figure 3, and the clutter signal c[n] can be expressed as:

(2) $$c_{k}\left[n\right]=\alpha {\;c}_{k-1}\left[n\right]+\left(1-\alpha \right)x_{k}\left[n\right]$$

Here, the term x[n] represents the received radar signal containing the series of received impulses, and constant α represents the weighting value ranging between 0 and 1. The constant α is a tradeoff between fast update and accuracy. For this paper, α was adjusted at 0.95, based on experimentation. Usually, for small movements, alpha should be adjusted close to 1. It can be observed in Equation (2) that the present clutter signal c[n], for the present input x[n] is estimated using both the input signal, and the previously estimated clutter. After estimating the clutter, it needs to be removed from the original signal. The final output signal y[n] of the clutter removal filter expressing the information of moving objects within the beam-width of radar can be written as:

(3) $$y_{\;k}\left[n\right]=x\left[n\right]-c_{\;k}\left[n\right]$$

**Figure 4:**
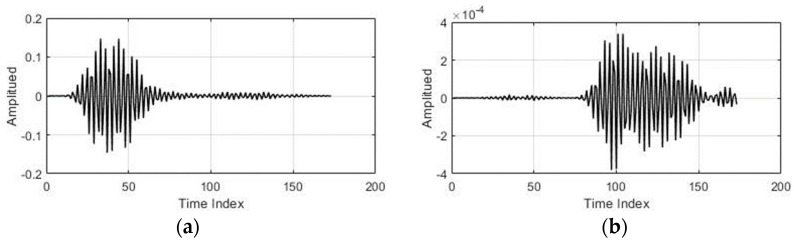
Gesture signal for single PRI: (**a**) before and (**b**) after clutter removal.

The average values of the fast-time indexes before and after clutter removal from one of the data samples (gesture 5) are presented in Figure 4a,b, i.e., averaged value of gesture signals at the input and the output of clutter removal filter respectively. A decrease in the output of the clutter-removed signal, which can be amplified, can be observed in the Figure 4. If we compare Figure 4a with Figure 4b then we can clearly notice that the clutter part of the signal, centered at sample 43 in Figure 4a is almost removed in Figure 4b using the loopback filter. The cluttered removed signal in Figure 4b contains only the gesture related part which is centered at sample 122. 

A sequence of short-duration pulses is transmitted by the radar, and the same sequence is repeated after a certain period known as the pulse repetition interval or (PRI) [31]. These repetitions are gathered in a 2D matrix, which can be represented as follows:

(4) $$r\left[n,m\right]=\overset{N}{\underset{n=1}{\sum }}y\left[n,k-m\right]+Noise$$

where *N* denotes the noise vector and *k* is the delay between the transmitted and received signals. The corresponding matrix form, known as the data matrix, can be written as follows:

(5) $$\vec{R}={\vec{Y}}_{\;n,\;m}+N$$

Here, rows ‘n’ and columns ‘m’ are referred to as the “fast time” and “slow time”, respectively. “Fast time” comprises a sequence of the pulses transmitted by the radar while “slow time” is the repetition of these transmitted pulses, depending upon the PRI.

The clutter-removed signal was statistically normalized before further processing. The objective of the statistical normalization process was to prepare a uniform set of data ranges for training purposes. Data normalization or feature scaling reduces the overall variance of the measurement data, making classification easier. For the purpose of normalization, we used the following equation:

(6) $${\vec{R}}_{\mathrm{normalized}}=abs\left(\frac{\vec{R}-µ}{\delta }\right)$$

where µ and δ are the mean and standard deviation of matrix $\vec{R}$. Here, we are subtracting the mean value of $\vec{R}$ from the original matrix and dividing the result by the overall standard deviation. The absolute of the resultant is taken to obtained the final normalized matrix. The normalization process was performed for each training sample.

### 2.4. Representation and Analysis of Gesture Data

**Figure 5:**
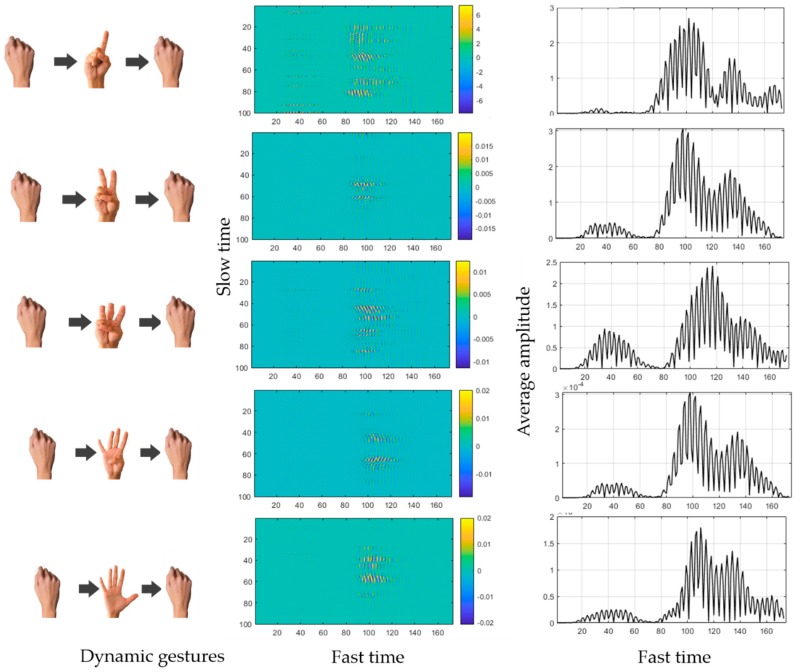
Dynamic gestures and corresponding generated 1 and 2-dimensional signals.

The gesture vocabulary used for counting purpose is represented in Figure 5. The first column of Figure 5 represents the dynamic gestures used for the purpose of counting whereas, second and third columns represent the respective two dimensional data matrix and one dimensional absolute averaged signal. The gestures are classified based on the “count” of the raised fingers. Data corresponding to finger count “one” appears to be noisier in comparison to finger count “five”. For the case of data matrix representation in second column, magnitude of received signal is represented as increasing color from blue to yellow. The signal pattern in a single dimension (when averaged for a certain slow time) is not as clearly distinguishable when compared to the variations between the patterns in two dimensions.

As depicted in Figure 5, it can be observed that there exist minute variations between all adjacent gestures. Hence, it was difficult to classify these gestures using one dimensional features. Therefore, we converted the signal representations into 2D images for further analysis. 

As stated above, given that the signal statistics did not allow for robust classification, we converted the signals into images using algorithm 1, for further analysis in two dimensions. 

**Figure 6:**
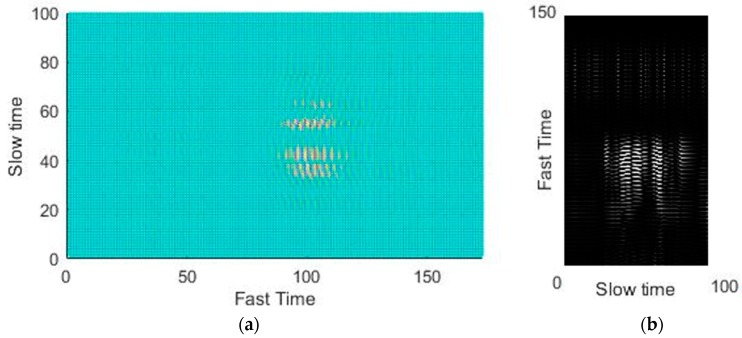
Image data corresponding to single gesture: (**a**) 2D data matrix and (**b**) corresponding greyscale image.

After normalization of the 2D data matrix, we transformed that matrix into an image with pixel values ranging from 0–255 as can be seen from Figure 6. The black color show pixel value of 0 and white color represent a value of 255. 

**Algorithm 1.** Transformation of radar signals into images
Receive input signal from radar sensor s(n).Remove clutter from signal as described in Section 2.3.Low-pass signals are combined into matrix of size m×n where “*m*” is slow-time index and “*n*” is fast-time index.Combine each one-dimensional signal from radar, as shown in Equation (5).Normalize database, as described by Equation (6).Convert normalized 2D matrices into RGB images and then convert it into greyscale images, as shown in Figure 6.


**Figure 7:**
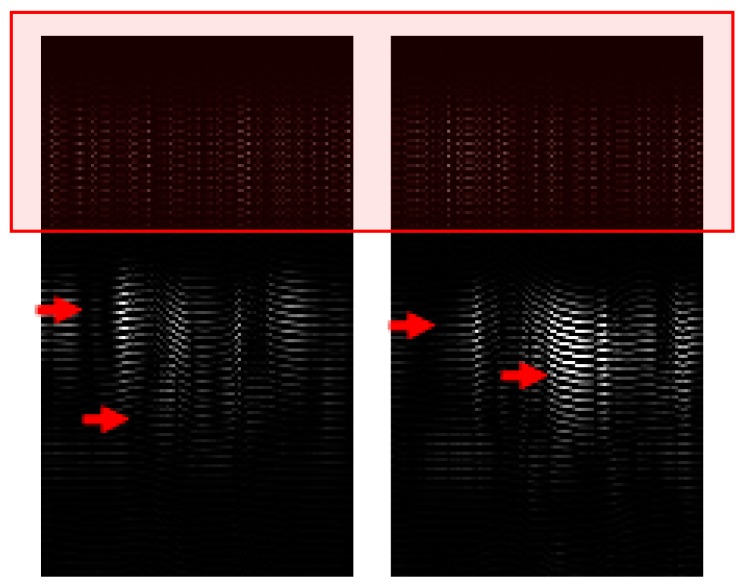
Similarities and differences in 2D gesture data.

Figure 7 shows the variations in the data generated using small finger movements. The boxed portion represents the area with similar patterns and the red arrows denote the differences in the patterns, which can be exploited using a sophisticated machine-learning-based classifier. 

### 2.5. CNN Architecture for Training and Classification

As is the case for any classification problem, the accuracy of classifying the desired patterns depends on the availability of high-quality spectral and temporal features. However, in the case of IR-UWB radar data, the features are usually time based as the frequency spectrum is broad [26]. Main advantage of CNN for IR-UWB radar is that it doesn’t require pre-extracted features. The algorithm extracts the features by itself. 

**Figure 8:**
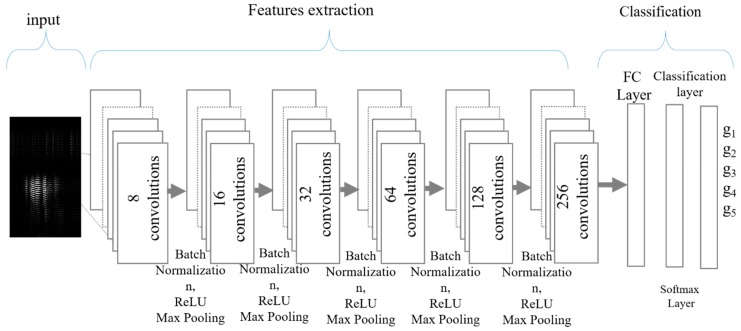
Architecture of implemented Convolutional Neural Network with 6 hidden layers.

The implemented CNN network is shown in Figure 8, which shows the different layers of the CNN for our classification problem. In accordance with the size of the radar’s data matrix, the input image dimensions for the CNN network were 173 × 100. For CNN, a smaller filter size is generally capable of extracting more detailed features, therefore a ‘3 × 3’ filter was used at each convolutional layer. To make all images have a balanced (normalized) distribution, zero-centered normalization is generally performed for the input layer. After each convolution layer, batch normalization is performed to speed up the training process [32]. Then a rectified linear unit (ReLU) layer serves as the activation function. For CNN networks, the ReLU layer is normally added after convolutional and batch normalization layers. Here at the output of ReLU, any value less than zero will be considered as zero as shown in Equation (7):

(7) $$h\left(x\right)=\{\begin{array}{c} x,\;x\geq 0\\ 0\;x\leq 0 \end{array}$$

Number of layers are increased linearly as a function of 2n and total six hidden layers were created for training and evaluation. The order of operation for each layer was: convolution, batch normalization, ReLu, and max pooling at the end. The high level and low level features were calculated while performing the convolutional operation and at the end fully-connected (FC) layer was made. Soft max function is performed after creating FC layer. Later cross-entropy based classification is performed to predict the gesture. The combination of all these layers collectively makes a network that mimics human brain up to some extent [33].

**Figure 9:**
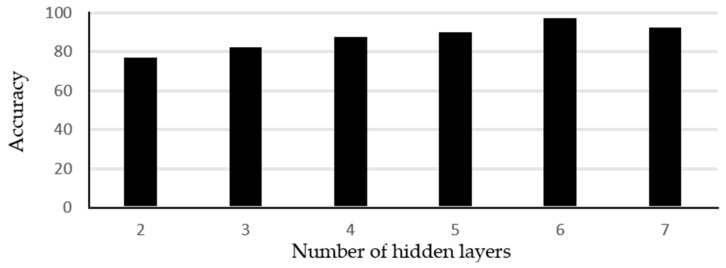
Accuracy as function of number of hidden layers in CNN.

In case of non-linearly separable data, the number of layers in he CNN architecture are normally selected based on trial and error methods [34]. The number of hidden layers affects the accuracy as well as processing time of training and evaluation. Fewer than the required hidden layers may provide an undertrained classifier whereas, too many hidden layers will cause the network to learn unnecessary details. We carefully performed a manual search process to optimize the CNN architecture, i.e., the network was trained with different number of hidden layers. Note that the network optimization was performed after selecting the preprocessing parameters and in case of any alteration in pre-processing block, the optimization process should be repeated again. Figure 9 represents the obtained test accuracy for different architectures of CNN. The optimization was performed by considering accuracy as a function of ‘number of hidden layers’. The layers were increased until we achieved maximum classification accuracy and minimum processing time for the given training dataset. As seen in Figure 9, the accuracy keeps on increasing with increase in number of layers. Even accuracy above 90% is observed with four and five layers as well. Maximum classification accuracy was observed with six hidden layers.

## 3. Experimental Setup

**Figure 10:**
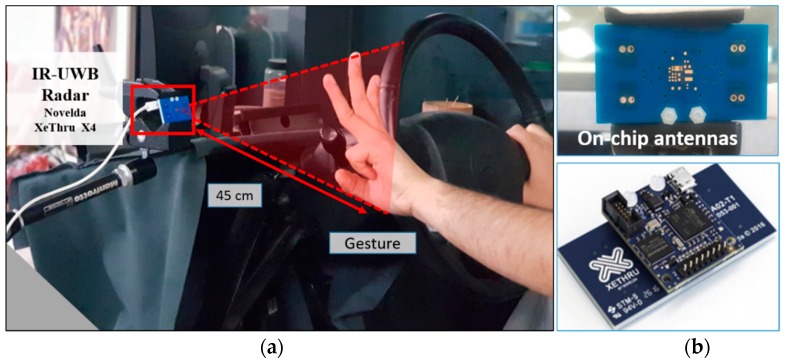
Hardware setup: (**a**) radar installed in car interior and (**b**) Novelda XeThru X4 radar sensor.

Figure 10a shows the experimental setup used for acquiring data and evaluating the performance of the proposed gesture system. The experiments were performed in laboratory environment; therefore, the interior of a car equipped with an IR-UWB radar was recreated to test the feasibility of the proposed system in a physical environment. Note that the radar was installed at point P1 in Figure 2. 

Figure 10b shows the radar sensor used for this experiment. A XeThru X4 (Novelda, Kviteseid, Norway) radar with an inbuilt transmitter and receiver antenna was employed in this study. The parameters of the radar transceiver are shown in Table 1. We used MatLab and the associated Deep Learning Toolbox for data acquisition, processing, and CNN architecture implementation. The radar sensor was connected to MatLab on the host computer via a serial protocol and the acquired data was pre-processed and converted into images. Further, the CNN classifier was built using the Deep Learning Toolbox of MatLab.

Table 2 shows the details of the implemented CNN design.

## 4. Results

### 4.1. Results of Clutter Removal Filter

**Figure 11:**
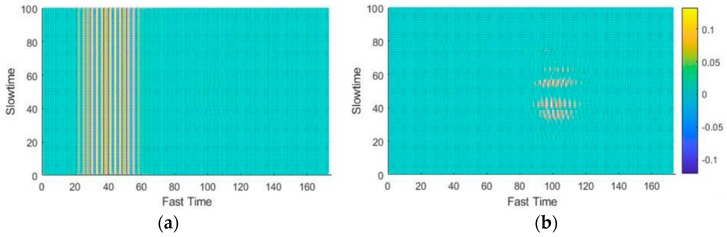
Clutter removal: (**a**) signal before removal and (**b**) signal after removal.

The acquired data matrix was first passed through a clutter-removal filter to remove unwanted echoes. The input and corresponding output of the clutter-removal filter are shown in Figure 11a,b, respectively. When the data passes through hidden layers, some spatial information is lost during the whole process [22]. As a result, minute shift in the pattern within the designated image frame will be negligible.

### 4.2. Optimal Sensor Position 

**Figure 12:**
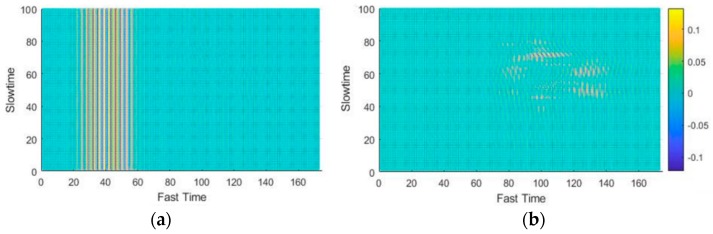
Clutter removal when radar placed near driver’s head: (**a**) signal before removal and (**b**) signal after removal.

In order to confirm the suitability of the selected sensor position, data were collected for two different radar positions. Figure 12a,b respectively show the input and output of the clutter-removal filter when the radar was placed at the top of the wind screen, that is, near the head of the driver (or position 2 in Figure 2). It was observed that, in this case, information related to the movements of the head was present even after the use of the clutter-removal filter (as seen in Figure 12b). At this location, signals related to the head movements get mixed with those related to the desired hand movements, making gesture recognition difficult. On the other hand, the placement of the radar at the side of the steering wheel effectively removed the clutter, with the filtered data being related only to the gestures (as seen in Figure 11b).

### 4.3. Gesture Image Patterns

**Figure 13:**
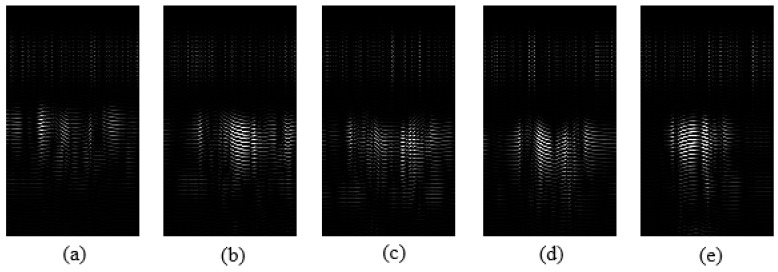
Images corresponding to individual gestures: (**a**) one, (**b**) two, (**c**) three, (**d**) four, and (**e**) five fingers.

The images corresponding to the individual gestures are presented in Figure 13. Moving from the right to the left, Figure 13a–e represent finger counts of one, two, three, four, and five respectively. Here, the brightness indicates the presences of a highly reflective object in the path of the radar beam width at the output of clutter removal filter. Figure 13e corresponding to gesture 5 contains a large number of white pixels in comparison with images generated corresponding to other gestures. 

### 4.4. Classification Results

**Figure 14:**
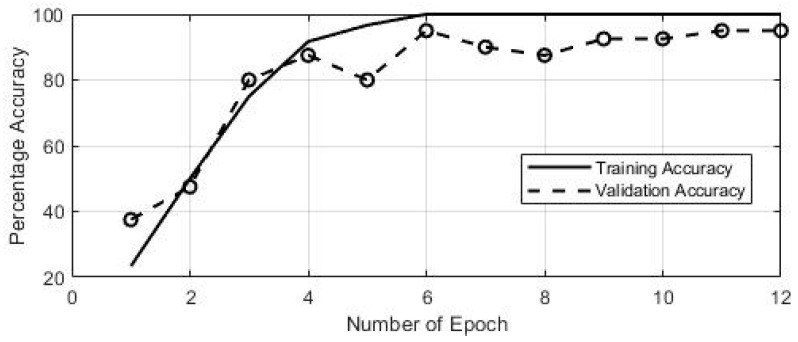
Training and validation accuracy of the CNN classifier.

Next, the above generated images were fed as an input to the CNN classifier for training and evaluation purposes. In the interest of robustness, the experiment was repeated using three different subjects, and 100 samples were gathered. We used 60% of the collected data for training purpose and the remaining 40% was used to evaluate the trained network. The training and validation accuracies are represented in Figure 14. The training accuracy is computed against each individual epoch and validation accuracy is found using test data. The average accuracy, rounded off to the nearest double digit, is listed in Table 3. As can be observed from the table, the CNN classifier distinguished 13% of gestures four as five. On the other hand, the gestures one, two, three and five were distinguished correctly. However, the classification accuracy can be increased by increasing the number of training samples, as any classification algorithm can train itself more efficiently using a larger training data set [21]. 

## 5. Conclusions

In this study, we have developed an algorithm for counting fingers based on gestures with the aim of controlling electronic devices in cars using these gestures. We used a single IR-UWB radar for gesture recognition. We first determined the optimal location for the radar within the car such that it would be convenient for the driver and the radar signal would not be subjected to interference from the undesirable motions of the driver arising from various driving activities. We performed the experiments in the area that is located in front of a driver which is at a short distance from the radar sensor. The one dimensional averaged signal for each gesture was not clearly distinguishable, therefore, we transformed the radar waveforms for a certain slow time into an image and then analyzed the patterns using those images with an image processing technique. The demonstration of mapping radar data into greyscale images was also demonstrated in this study. CNN algorithm was used for feature extraction and classification. No feature set was provided as CNN algorithm extract features by itself. The prediction accuracy was high for all five gestures. In the future, we aim to develop an algorithm that can recognize the finger counts of two hands, so that a total of ten gestures can be recognized using a single radar sensor. 

## Figures and Tables

**Table 1:** Radar transceiver parameters.

Parameter	Value
Output power	−12.6 dBm
Center frequency	8.748 GHz
Pulse repetition frequency	100 MHz
Bandwidth (−10 dB)	2.3 GHz
Sampling frequency	23 samples/s
Staggered PRF sequence length	2^20^ cycles

**Table 2:** Hyper parameters values of implemented convolutional neural network.

Hyperparameter	Description
Number of hidden layers in CNN	06
Convolution filter size	03
Learning rate	0.01
Epochs	10

**Table 3:** Confusion matrix for experimental results.

	Predicted Gesture Class					
**Original Gesture Class**	**Gesture Class**	**One**	**Two**	**Three**	**Four**	**Five**
	**One**	1	0	0	0	0
	**Two**	0	1	0	0	0
	**Three**	0	0	1		0
	**Four**	0	0	0	0.87	0.13
	**Five**	0	0	0	0	1
